# Paracrine SDF-1α signaling mediates the effects of PSCs on GEM chemoresistance through an IL-6 autocrine loop in pancreatic cancer cells

**DOI:** 10.18632/oncotarget.3099

**Published:** 2014-12-30

**Authors:** Hui Zhang, Huanwen Wu, Jian Guan, Li Wang, Xinyu Ren, Xiaohua Shi, Zhiyong Liang, Tonghua Liu

**Affiliations:** ^1^ Department of Pathology, Peking Union Medical College Hospital, Chinese Academy of Medical Science, Beijing, PR China; ^2^ Department of Pathology, Cancer Institute and Hospital, Chinese Academy of Medical Sciences, Beijing, PR China

**Keywords:** Pancreatic cancer, Pancreatic stellate cells, Chemoresistance, SDF-1α, IL-6

## Abstract

Pancreatic cancer exhibits the poorest prognosis among all tumors and is characterized by high resistance to the currently available chemotherapeutic agents. Our previous studies have suggested that stromal components could promote the chemoresistance of pancreatic cancer cells (PCCs). Here, we explored the roles of pancreatic stellate cells (PSCs) and the SDF-1α/CXCR4 axis in pancreatic cancer chemoresitance. Our results showed that primary PSCs typically expressed SDF-1α, whereas its receptor CXCR4 was highly expressed in PCCs. PSC-conditioned medium (PSC-CM) inhibited Gemcitabine (GEM)-induced cytotoxicity and apoptosis in the human PCC line Panc-1, which was antagonized by an SDF-1α neutralizing Ab. Recombinant human SDF-1α (rhSDF-1α) increased IL-6 expression and secretion in Panc-1 cells in a time and dose-dependent manner, and this effect was suppressed by the CXCR4 antagonist AMD3100. rhSDF-1α protected Panc-1 cells from GEM-induced apoptosis, and the protective effect was significantly reduced by blocking IL-6 using a neutralizing antibody. Moreover, rhSDF-1α increased FAK, ERK1/2, AKT and P38 phosphorylation in Panc-1 cells, and either FAK or ERK1/2 inhibition suppressed SDF-1α-upregulated IL-6 expression. SDF-1α-induced AKT activation was almost completely blocked by FAK inhibition. In conclusion, we demonstrate for the first time that PSCs promote the chemoresistance of PCCs to GEM, and this effect is mediated by paracrine SDF-1α/CXCR4 signaling-induced activation of the intracellular FAK-AKT and ERK1/2 signaling pathways and a subsequent IL-6 autocrine loop in PCCs. Our findings indicate that blocking the PSC-PCC interaction by inhibiting SDF-1α/CXCR4 signaling may be a promising therapeutic strategy for overcoming chemoresistance in pancreatic cancer.

## INTRODUCTION

Pancreatic cancer is a highly lethal malignancy with occult symptoms, rapid development, early metastasis and high resistance to chemotherapy [1, 2]. At the time of diagnosis, the tumor is confined to the pancreas in only approximately 10% of patients. The majority of patients have locally advanced tumors and/or unresectable distant metastases [3]. Therefore traditional chemotherapy is indispensable in the current treatment of pancreatic cancer. However, even the most effective first-line chemotherapeutic agent, gemcitabine (GEM), can only achieve a slight improvement in survival. Moreover, numerous clinical studies have shown that the combination of GEM with other cytotoxic drugs is unlikely to significantly improve the prognosis of pancreatic cancer. Therefore, there is an urgent need to develop novel therapeutic strategies to effectively treat this devastating carcinoma [4, 5].

Pancreatic stellate cells (PSCs) are a crucial component in the tumor microenvironment of pancreatic cancer [6]. PSCs remain in a quiescent state in normal pancreas, however, they can transition to a myofibroblast-like phenotype under various pathological conditions, including cancer and inflammatory disease. Activated PSCs are able to proliferate, migrate, and synthesize various cytokines and extracellular matrix components, resulting in extensive proliferation of fibrous connective tissue (desmoplasia), one of the most characteristic histopathological changes in pancreatic cancer [7-9]. In recent years, researchers have increasingly recognized the critical role of the interaction between PSCs and pancreatic cancer cells (PCCs) in pancreatic cancer pathobiology. Tumor cells secrete extracellular factors to activate PSCs, and in turn, PSCs release a large number of cytokines and extracellular matrix proteins, regulating tumor cell proliferation, apoptosis, invasion, metastasis, and drug resistance [8-10]. Our recent studies have also revealed that PSCs and the stromal microenvironment play a critical role in pancreatic cancer development and chemoresistance and indicated that blocking the stroma-tumor cell interaction may be a promising therapeutic strategy for overcoming chemoresistance in pancreatic cancer [11, 12].

Stromal cell-derived factor-1 (SDF-1), also named CXCL12 (chemokine (C-X-C motif) ligand 12)), is a member of the CXC chemokine family of pro-inflammatory mediators. SDF-1 exerts its biological effect by binding and activating the CXC chemokine receptor 4 (CXCR4), a G protein-coupled receptor, which is the predominant receptor for SDF-1 and is frequently overexpressed in a variety of human cancer cells. As the predominant isoform of SDF-1, SDF-1α is expressed in many organs [13, 14]. In recent years, it has become apparent that the SDF-1α/CXCR4 biological axis is a critical mediator of tumor–stromal interactions and is closely related to the malignant process and poor prognosis in a variety of epithelial cancers, such as pancreatic cancer, liver cancer, lung cancer, breast cancer, and prostate cancer [15-19]. Although preliminary data have indicated that the SDF-1/CXCR4 axis might induce chemoresistance in PCCs, its underlying mechanism remains largely unknown. In addition, it is unclear whether and how the SDF-1/CXCR4 axis mediates PSC-induced chemoresistance in pancreatic cancer.

In the present study, we investigated the roles and mechanisms of PSCs and the SDF-1α/CXCR4 biological axis in GEM chemoresistance in pancreatic cancer. Our study aimed to further clarify the mechanism of chemoresistance in a tumor microenvironment-dependent model and identify novel therapeutic targets for overcoming chemoresistance in pancreatic cancer.

## RESULTS

### SDF-1α and CXCR4 expression in PSCs and PCCs

**Figure 1:**
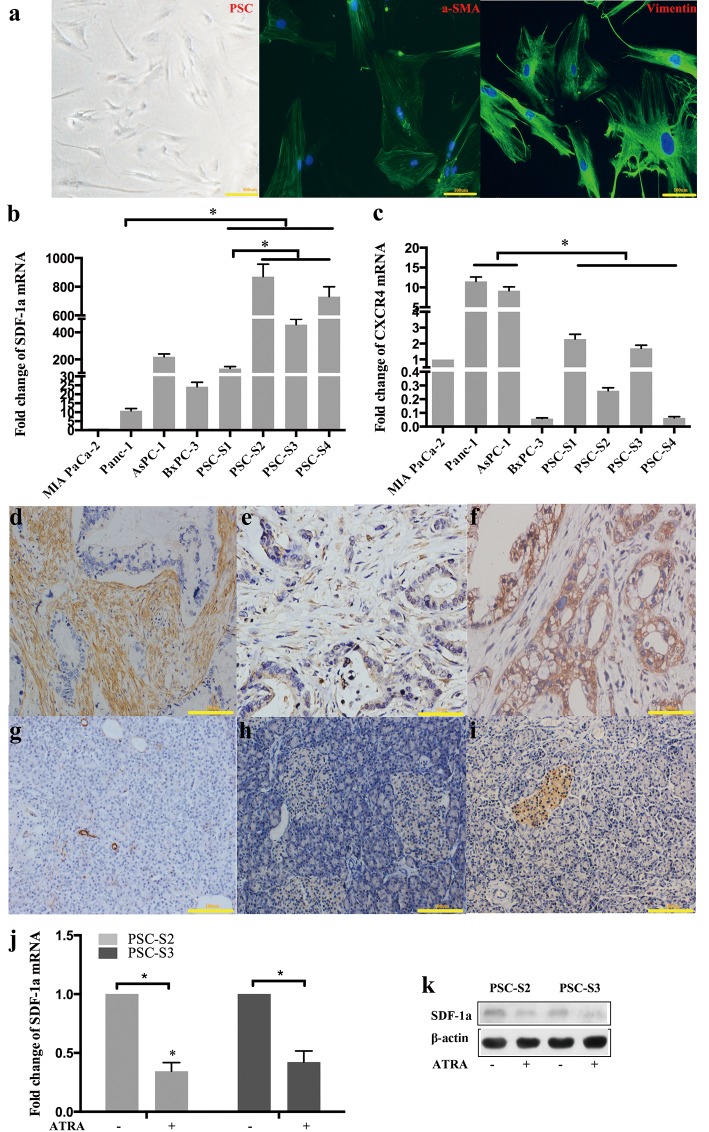
SDF-1α and CXCR4 expression in PSCs and PCCs Activated primary PSCs isolated from pancreatic cancer tissues were verified by immunofluorescence staining for α-SMA and vimentin (a); SDF-1a mRNA (b) and CXCR4 (c) mRNA expression was examined by RT-qPCR. The expression of the target mRNA was normalized to that of β-actin mRNA. The values are expressed relative to 1.00 for expression in MIA PaCa-2 cells. The bars represent the mean of three independent experiments ± SE. *, P<0.05. In the four resected pancreatic cancer samples used for PSC isolation, α-SMA, SDF-1α and CXCR4 were detected by immunohistochemistry in pancreatic cancer tissues (d, e and f) and distant normal pancreatic tissues (g, h and i). Two primary PSCs were harvested and analyzed for SDF-1α expression by RT-qPCR (j) and Western blot (k).

Activated primary PSCs isolated from pancreatic cancer tissues were verified by immunofluorescence staining for α-SMA and vimentin (Figure 1a). We evaluated the mRNA expression level of SDF-1α and CXCR4 in four PCC lines (MIA PaCa-2, Panc-1, AsPC-1, BxPC-3) and four primary PSCs (PSC-S1, PSC-S2, PSC-S3, PSC-S4) by RT-qPCR. SDF-1α mRNA expression in the four PSCs was significantly higher than that in Panc-1, MIA PaCa-2 and BxPC-3 cells. Among the four PSCs, PSC-S1 showed a relatively lower level of SDF-1α mRNA expression (Figure 1b). In contrast with SDF-1α, CXCR4 mRNA expression in the four PSCs was significantly lower than that in Panc-1 and AsPC-1 cells (Figure 1c). Because of the expression pattern, Panc-1 cells (low SDF-1α expression and high CXCR4 expression) were used for the subsequent experiments on the PSC-PCC interaction.

We also investigated a-SMA, SDF-1α and CXCR4 protein expression in the four resected specimens used for PSCs isolation by immunohistochemistry (Figure 1d-1f). Activation of the PSCs in the pancreatic cancer tissues was confirmed by the expression of a-SMA. In all four cases, the PCCs demonstrated moderate to strong CXCR4 staining and weak SDF-1α staining, while PSCs in three cases (PSC-S2, PSC-S3, PSC-S4) showed moderate to strong SDF-1α staining and negative CXCR4 staining. However, PSC-S1 was negative for both SDF-1α and CXCR4 staining. Given the relatively low expression level of SDF-1α in PSC-S1, we used the other three PSCs to harvest PSC-CM for further investigation.

We also found that distant samples of normal pancreas tissue in all cases showed negative staining for a-SMA, SDF-1α and CXCR4 (except islet cells) (Figure 1g-1i). To further confirm whether the high SDF-1α expression was due to PSCs activation in pancreatic cancer, we induced activated PSCs to enter a relatively quiescent state by treating the cells with all-trans retinoic acid (ATRA). ATRA is an active metabolite of vitamin A. Our previous studies showed that ATRA could prevent the activation of PSCs by decreasing cell proliferation, a-SMA expression and collagen production [12]. After treatment with ATRA, the PSCs showed morphological changes and contained fat droplets, similar to quiescent PSCs (data not shown). We found that the levels of SDF-1α mRNA and protein in PSCs were significantly decreased after ATRA treatment (Figure 1j and 1k).

### SDF-1α mediated the effects of PSCs on GEM-induced cytotoxicity in Panc-1 cells

**Figure 2:**
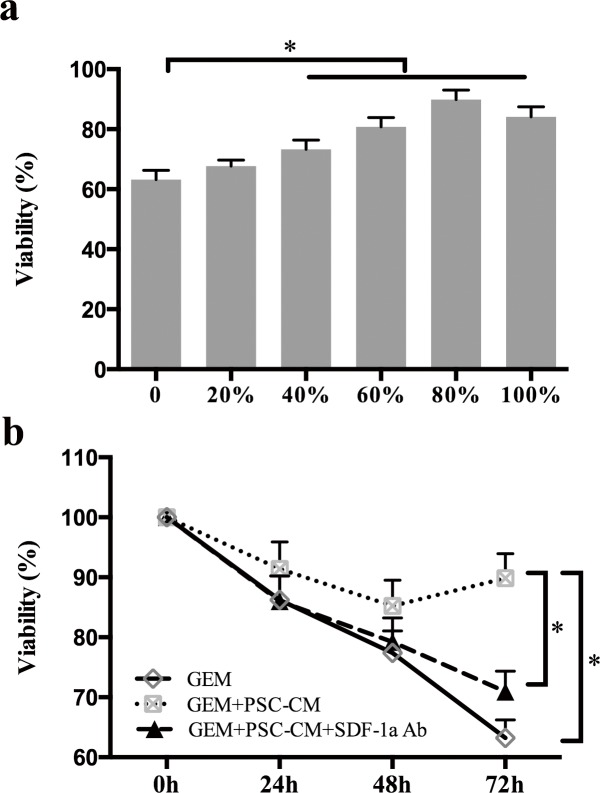
PSC-CM suppressed GEM-induced cytotoxicity in Panc-1 cells (a): Panc-1 cells were treated with GEM (10 μM) in the presence of various doses of PSC-CM. Cancer cell viability was examined 72 h post-treatment using the MTT assay. (b): Panc-1 cells were treated with GEM (10 μM) with or without PSC-CM (80%) and SDF-1a Ab (100 ng/ml). After 24 h, 48 h, or 72 h of treatment, cell growth was determined using MTT. Bars represent the mean of three independent experiments ± SE.*, P<0.05.

To clarify whether PSCs can mediate the chemoresistance of PCCs, Panc-1 cells were treated with GEM in the presence of various doses of PSC-CM. The MTT assay showed that PSC-conditioned medium (PSC-CM) inhibited GEM-induced cytotoxicity in Panc-1 cells in a dose-dependent manner (Figure 2a and 2b). High doses of PSC-CM (40% or more) significantly decreased GEM-induced cytotoxicity in Panc-1 cells (P <0.05). The Panc-1 cell viability reached a peak when the cells were co-cultured with 80% PSC-CM, although no significant difference was found between Panc-1 cells co-cultured with 80% and 100% PSC-CM.

Subsequently, Panc-1 cells were cultured in serum-free medium containing 80% PSC-CM and then treated with GEM in the absence or presence of an SDF-1α neutralizing Ab for 24 h, 48 h, or 72 h. The MTT assay demonstrated that the protective effect of PSCs on GEM-induced cytotoxicity was significantly antagonized by specific inhibition of SDF-1a in a time-dependent manner (Figure 2b).

### SDF-1α mediated the effects of PSCs on GEM-induced apoptosis in Panc-1 cells

Apoptosis is considered to be the major mechanism of GEM-induced cell death [11]. To further explore the roles of PSCs and SDF-1α in the chemoresistance of Panc-1 cells, the effects of PSC-CM and SDF-1α neutralizing Ab on GEM-induced apoptosis in Panc-1 cells were assessed by Hoechst staining and Annexin V/PI flow cytometry analysis.

**Figure 3:**
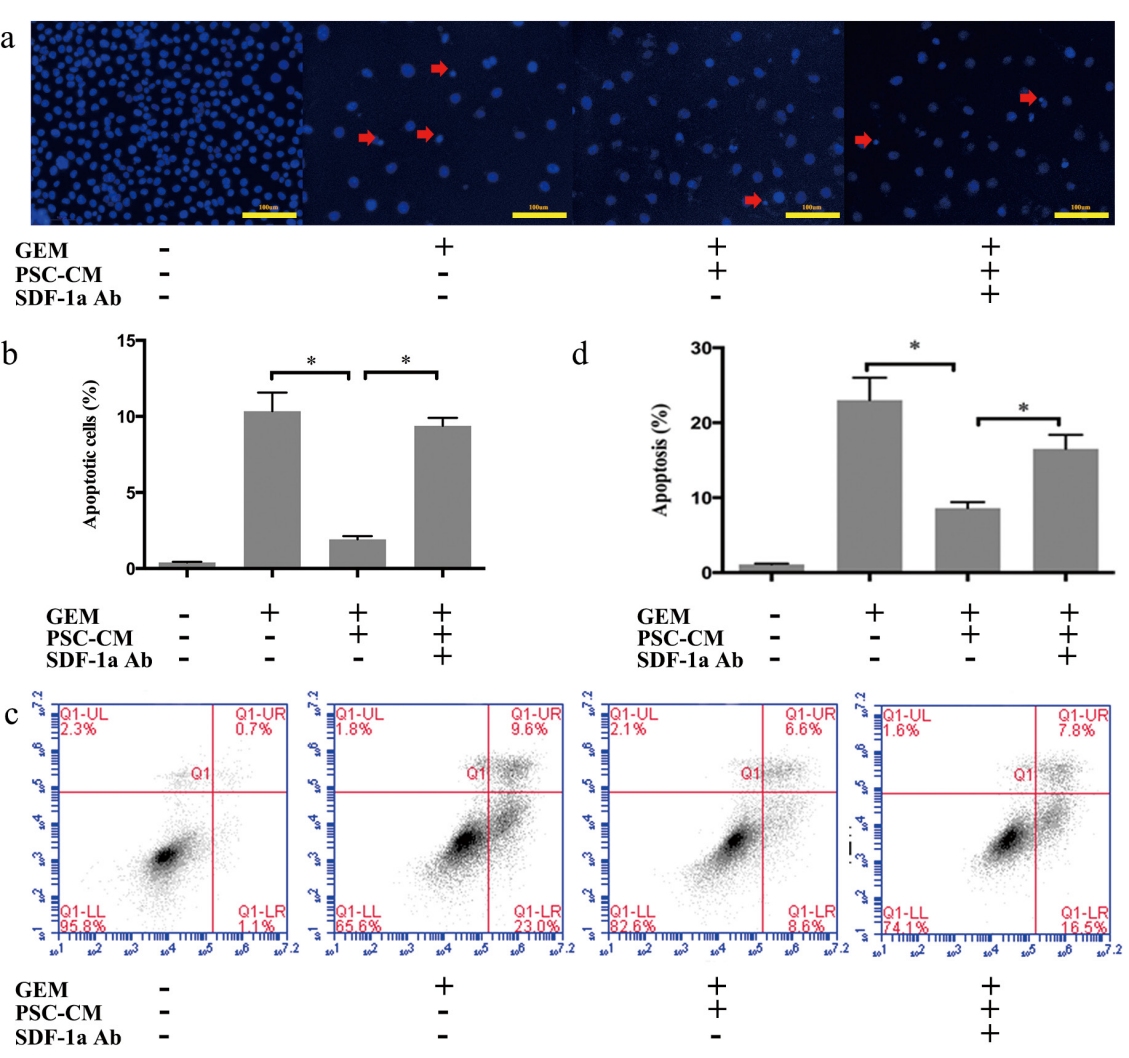
SDF-1α mediated the effects of PSC-CM on GEM-induced apoptosis in Panc-1 cells Panc-1 cells were treated with GEM (10 μM) with or without PSC-CM (80%) and SDF-1α Ab (100 ng/ml) for 72 h. (a): Apoptotic cells were examined by Hoechst 33342 staining (the arrow indicates the apoptotic cells), and (b) the percentage of apoptotic cells statistically analyzed. (c): Cells were harvested for flow cytometry analysis of Annexin-V/PI labeling, and (d) the proportion of cells in early-stage apoptosis was statistically analyzed. Bars represent the mean of three independent experiments ± SE. *, P<0.05.

Hoechst staining indicated that the number of GEM-induced apoptotic Panc-1 cells was significantly decreased by PSC-CM, which was demonstrated by an decreased proportion of condensed nuclei (P<0.05) (Figure 3a and 3b). Moreover, specific inhibition of SDF-1α by a neutralizing Ab antagonized the protective effect of PSC-CM on GEM-induced apoptosis in Panc-1 cells (P<0.05). Similar results were observed with Annexin V/PI analysis (Figure 3c and 3d). PSC-CM significantly decreased the early apoptosis rate induced by GEM from 23% to 8.6% (P<0.05), which was at least partially blocked by specific inhibition of SDF-1α (P<0.05). Collectively, these findings suggested that SDF-1α at least partially mediated the effects of PSC on GEM-induced apoptosis in Panc-1 cells.

### The SDF-1α/CXCR4 axis upregulated IL-6 expression in Panc-1 cells

As a chemotactic factor, SDF-1α has been reported to stimulate endothelial cells, fibroblasts and tumor cells to secrete a variety of factors, such as IL-6, IL-8, and matrix metalloproteinases (MMP). We further explored the effects of SDF-1α on IL-6 and IL-8 expression in Panc-1 cells.

**Figure 4:**
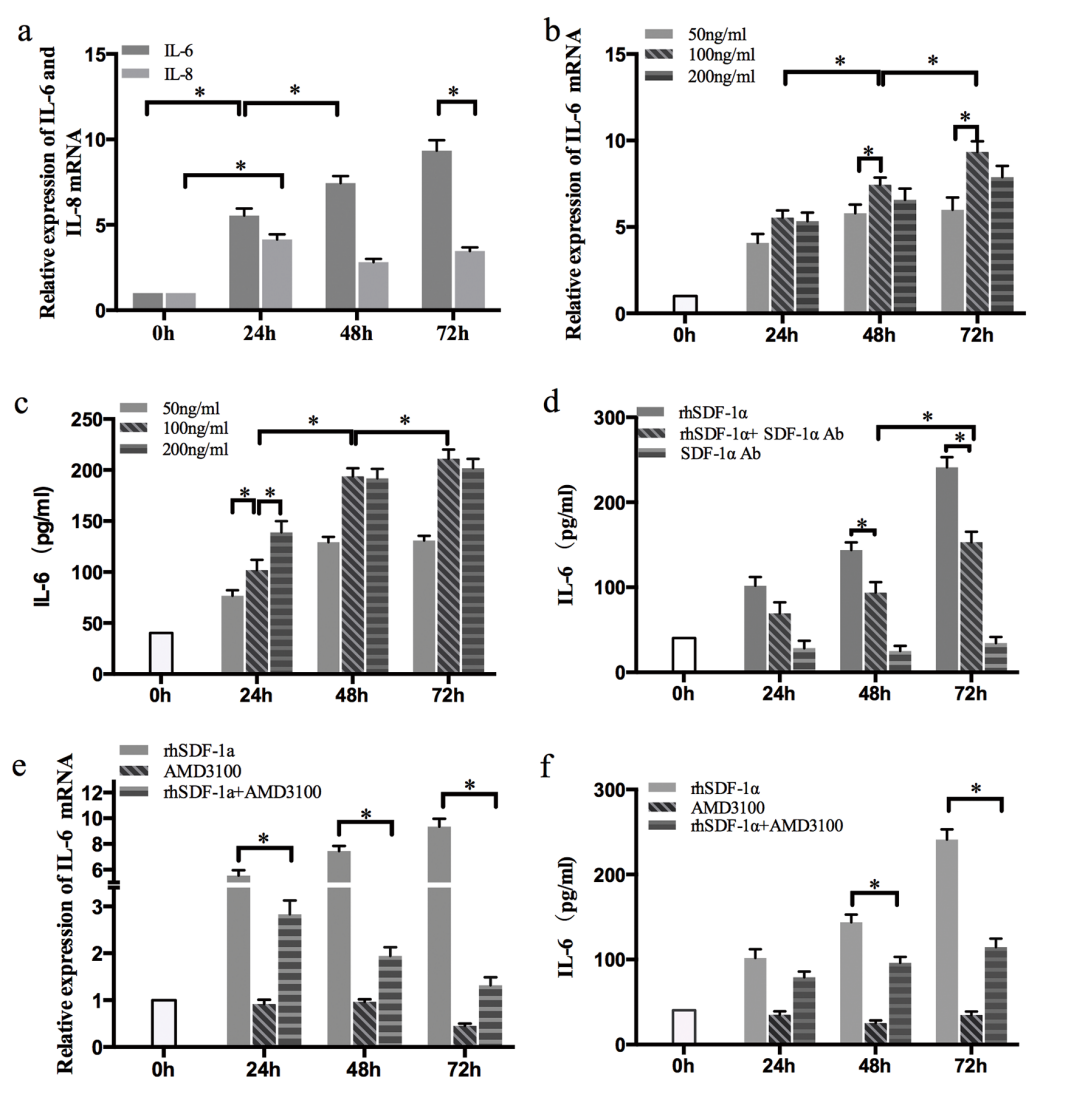
The effects of SDF-1α on GEM chemoresistance in Panc-1 cells were mediated by IL-6 (a): Panc-1 cells were treated with rhSDF-1α protein (100 ng/ml) or control vehicle, cultured for 24 h, 48 h, or 72 h and harvested for RT-qPCR of IL-6 and IL-8. (b): Cells were treated with various concentrations of rhSDF-1a for 24 h, 48 h, or 72 h, and IL-6 mRNA was examined by RT-qPCR. (c): Cells were treated with various concentration of SDF-1α for 24 h, 48 h, or 72 h. IL-6 protein was examined by ELISA. (d): Cells were treated with rhSDF-1α (100 ng/ml) and/or SDF-1α Ab (100 ng/ml) for 24 h, 48 h, or 72 h, and IL-6 protein was examined by ELISA. Panc-1 cells were pretreated with the CXCR4 receptor antagonist AMD3100 or the vehicle for 6 hours. Cells were treated with rhSDF-1α (100 ng/ml) or the vehicle for an additional 24 h, 48 h, of 72 h. The cells and supernatants were collected for analysis of IL-6 by RT-qPCR (e) and ELISA (f), respectively. The bars represent the mean of three independent experiments ± SE. *, P < 0.05.

Panc-1 cells were treated with rhSDF-1α (100 ng/ml) for 0 h, 24 h, 48 h, 72 h and harvested for RT-qPCR. The mRNA levels of both IL-6 and IL-8 were increased significantly (*P*<0.05) (Figure 4a and 4b). However, compared with IL-8, the IL-6 mRNA level was more significantly altered and showed a time-dependent pattern with a peak at 72 h. To further test whether rhSDF-1α induced IL-6 expression in a dose-dependent manner, Panc-1 cells were treated with different concentrations of rhSDF-1α as indicated in Figure 4c and 4d, and the IL-6 expression and secretion levels were detected by RT-qPCR and ELISA, respectively. Our results revealed that rhSDF-1α increased IL-6 expression and secretion in Panc-1 cells in both a time-dependent and a dose-dependent manner, with a peak value in cells treated with 100 ng/ml rhSDF-1α for 72 h.

In recent years, it has been reported that SDF-1α can also exert its biological effect by binding to another CXC chemokine receptor, CXCR7. Moreover, gene regulation via SDF-1α/CXCR4 and SDF-1α/CXCR7 may be completely different [21]. Therefore, we confirmed whether the upregulation of IL-6 by SDF-1α in Panc-1 cells was mediated by CXCR4. Our results showed that the SDF-1α-induced upregulation of IL-6 expression was significantly suppressed by the CXCR4 antagonist, AMD3100 (Figure 4e and 4f).

### The enhancing effects of SDF-1α on GEM chemoresistance in Panc-1 cells were mediated by IL-6

**Figure 5:**
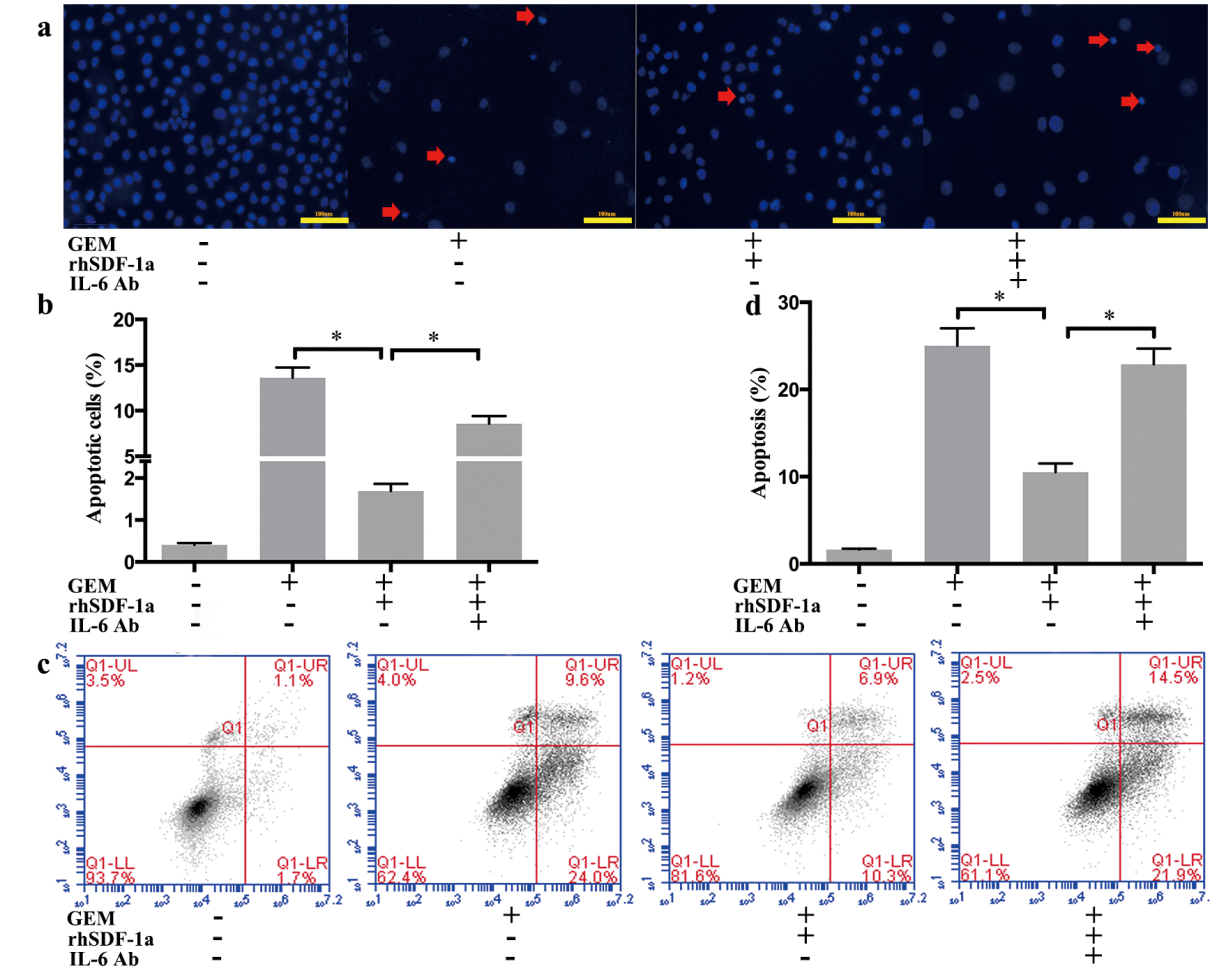
SDF-1α mediated the effects of PSCs on GEM-induced apoptosis in Panc-1 cells Panc-1 cells were cultured in serum-free medium containing GEM (10 μM), with rhSDF-1α (100 ng/ml), IL-6 Ab (1 μg/ml) or the vehicle for 72 h. (a): Apoptotic cells were examined by Hoechst 33342 staining (the arrow indicates the apoptotic cells), and (b) the percentage of apoptotic cells was statistically analyzed. (c): Cells were harvested for flow cytometry analysis of Annexin-V labeling, and (d) the proportion of cells in early-stage apoptosis was statistically analyzed. Bars represent the mean of three independent experiments ± SE. *, P < 0.05.

Previous studies have shown that the serum levels of IL-6 were elevated and negatively correlated with the therapeutic effects of GEM in patients with pancreatic cancer [22]. As described above, we verified that SDF-1α mediated the effects of PSC-CM on GEM chemoresistance, and exogenous SDF-1α led to the upregulation of IL-6 expression in Panc-1 cells. Therefore, we further explored the influence of IL-6 on GEM-induced apoptosis in Panc-1 cells. Our results showed that the protective effect of rhSDF-1α on GEM-induced apoptosis was significantly inhibited by the specific inhibition of IL-6 using a neutralizing antibody (P<0.05) (Figure 5a-5d).

### SDF-1α upregulated IL-6 expression in Panc-1 cells through activation of the FAK-AKT and ERK1/2 signaling pathways

**Figure 6:**
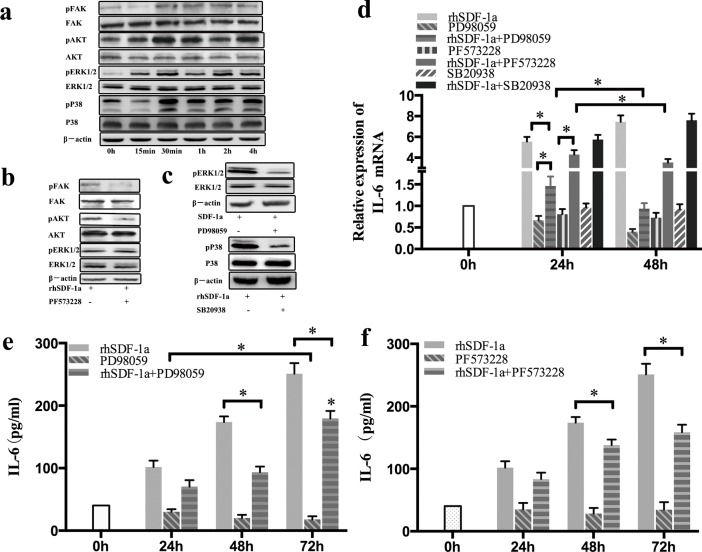
SDF-1α upregulated IL-6 expression in Panc-1 cells through the activation of FAK-AKT and ERK1/2 signaling (a): Panc-1 cells were treated with rhSDF-1α (100 ng/ml) or the vehicle for 15 min, 30 min, 1 h, 2 h or 4 h. The expression and phosphorylation of FAK, AKT, ERK1/2, and P38 in Panc-1 cells was detected by western blotting. (b): Panc-1 cells were pretreated with PF537228 (FAK inhibitor) or control vehicle for 1 h and then treated with rhSDF-1α for an additional 30 min. The expression and phosphorylation of FAK, AKT, and ERK1/2 in Panc-1 cells were examined by western blotting. (c): Panc-1 cells were pretreated with PD98059 (an ERK inhibitor), SB20938 (a P38 inhibitor) or the vehicle for 1 h, followed by treatment with rhSDF-1α for an additional 30 min. The expression and phosphorylation of ERK1/2 and P38 in Panc-1 cells were detected by western blotting. (d): Panc-1 cells were pretreated with PF537228, PD98059, SB20938 or the vehicle for 1 h, followed by treatment with rhSDF-1α for an additional 24 h, 48 h, or 72 h. IL-6 mRNA expression in Panc-1 cells was tested by RT-qPCR. Panc-1 cells were pretreated with PD98059 (e), PF573228 (f) or vehicle for 1 h, followed by treatment with rhSDF-1a for an additional 24 h, 48 h, or 72 h. IL-6 expression in the culture supernatants was tested by ELISA. The bars represent the mean of three independent experiments ± SE. *, P < 0.05.

To further clarify the mechanism underlying the upregulation IL-6 by SDF-1α, we detected the expression and phosphorylation of FAK, AKT, ERK1/2 and P38 in Panc-1 cells by Western blot. Our results indicated that the levels of FAK, AKT ERK1/2 and P38 phosphorylation in Panc-1 cells were all significantly elevated after 30 min of rhSDF-1α treatment, and the effects lasted for 4 h. The levels of protein expression were not significantly affected (Figure 6a).

Both AKT and ERK1/2 are key kinases that act downstream of FAK. Our previous study demonstrated that the FAK/AKT signaling pathway is involved in pancreatic cancer chemoresistance [11]. Therefore, we further explored whether FAK functioned upstream of the AKT or ERK1/2 signaling pathways. The effect of SDF-1α on AKT activation in Panc-1 cells was almost completely blocked by specific inhibition of FAK phosphorylation using the small molecule inhibitor PF573228. However, SDF-1α-induced ERK1/2 phosphorylation in Panc-1 cells was not affected by FAK inhibition (Figure 6b).

To determine whether SDF-1α-induced FAK/AKT, ERK1/2 and P38 activation was involved in IL-6 upregulation in Panc-1 cells, small molecule inhibitors of FAK (PF573228), ERK1/2 (PD98059) and P38 (SB20938) were used. The effects of these inhibitors on their corresponding kinase activity were verified by western blot (Figure 6b-6c). SDF-1α-induced upregulation of IL-6 mRNA and protein expression was partially antagonized by both FAK/AKT inhibition and ERK1/2 inhibition (P<0.05) in a time-dependent manner (Figure 6e and 6f), while specific inhibition of P38 activation did not affect the upregulation of IL-6 expression induced by SDF-1α.

These results confirmed that SDF-1α upregulates IL-6 expression through the activation of the FAK-AKT and ERK1/2 signaling pathways, and not the P38 signaling pathway, in Panc-1 cells.

## DISCUSSION

A high resistance to chemotherapy and prominent desmoplastic stroma are two main contributing factors to the poor outcome of pancreatic cancer. Our group and others have increasingly recognized the critical role of tumor stroma in pancreatic cancer chemoresistance [11, 23, 24]. PSCs are a critical cellular component of pancreatic cancer stroma. In pancreatic cancer, PSCs transition from a quiescent phase to an activated phase in which they proliferate, migrate, and secrete excessive amounts of soluble factors and extracellular matrix (ECM) proteins, leading to fibrosis of the pancreas. Activated PSCs may regulate PCCs growth, survival, metastasis, and chemoresistance [8, 9, 25]. However, the mechanism of chemoresistance mediated by PSCs in pancreatic cancer is not well defined.

SDF-1α and its specific receptor CXCR4 are highly expressed in a variety of tumors, including gastric, colorectal, breast, and ovarian cancers [26-29]. Our *in vivo* and *in vitro* results demonstrated that activated primary PSC from pancreatic cancer tissues typically expressed high levels of SDF-1α, while high CXCR4 expression was typically observed in PCCs. Moreover, distant normal pancreas tissue was negative for both SDF-1α and CXCR4 staining, and PSCs inactivation by ATRA significantly decreased SDF-1a expression in PSCs. Our results indicated that the SDF-1α/CXCR4 axis was activated in PCCs.

Previous studies have shown that PSC-CM could promote the proliferation, migration, invasion and organ-specific metastasis of PCCs through the SDF-1α/CXCR4 axis [30, 31]. Although the effect of the SDF-1α/CXCR4 axis on chemoresistance to GEM in PCCs has been reported [32], it remains unclear whether and how the SDF-1α/CXCR4 axis mediates the effect of PSCs on GEM chemoresistance. The present study confirmed that PSCs promoted the chemoresistance of PCCs to GEM, which was at least partially mediated by paracrine SDF-1α signaling. Our findings suggested that blocking the PSC-PCC interaction by inhibiting the SDF-1α/CXCR4 signaling pathways may be a promising therapeutic strategy for overcoming chemoresistance in pancreatic cancer. Compared with previous studies that focused on PCCs themselves, investigation of the interactions between PSCs or tumor stroma and tumor cells might provide more information about the conditions *in vivo* and thus be highly useful for studying chemoresistance. In our previous study, we reported that the ECM component laminin increased the chemoresistance of PCCs to GEM [11]. The results provided new insights into the critical role of the tumor microenvironment in chemoresistance and offered a reasonable explanation for the frequently observed discrepancy between *in vitro* drug sensitivity and the *in vivo* clinical response in pancreatic cancer.

SDF-1 has been reported to stimulate different types of cells to produce a variety of soluble factors, including IL-6, IL-8, and MMP, upon binding to its specific receptor CXCR4. Moreover, the effects are cell-type specific [33-35]. The present study found that the SDF-1α/CXCR4 axis upregulated IL-6 in a time- and dose-dependent manner in Panc-1 cells. As a multifunctional growth factor, IL-6 was originally discovered in T cells, and it can promote B cell maturation. In addition to the inflammatory response, IL-6 has also been shown to be associated with various biological behaviors of tumor cells, including growth, survival, metastasis, angiogenesis, epithelial-mesenchymal transformation (EMT) and chemoresistance [36-38]. *In vitro* and *in vivo* experiments confirmed that IL-6 could inhibit apoptosis of pancreatic intraepithelial neoplasia (PanIN) cell lines and promote the development of precancerous lesions and pancreatic cancer, which indicated that IL-6 is involved in early stages of pancreatic cancer development [39]. Mitsunaga [22] et al. reported that a high serum IL-6 level was a poor prognostic factor for overall survival in in patients with pancreatic cancer. Moreover, high expression of IL-6 receptor (IL-6R) was confirmed in PCCs and the activation of IL-6R-related pathway in tumor cells was associated with a poor outcome in resected pancreatic ductal adenocarcinoma [40, 41]. Our previous study also showed that IL-6 could prevent PCCs migration and the EMT [12]. All these results suggest that IL-6–mediated intracellular signaling cascades in tumor cells might play important roles in pancreatic cancer progression. In the present study, we revealed for the first time that exogenous SDF-1α induced intracellular expression and extracellular secretion of IL-6 in Panc-1 cells, and the protective effects of SDF-1α on GEM-induced apoptosis in Panc-1 cells were at least partially mediated by IL-6, indicating that an IL-6 autocrine loop might contribute to SDF-1α-induced GEM chemoresistance in PCCs. The IL-6 autocrine loop in PCCs might be an effective target for blocking SDF-1α-mediated PSC-PCC interactions. Autocrine IL-6 signaling has also been found to promote growth, metastasis and chemoresistance in a variety of tumors, including breast, ovarian and endometrial cancers [40, 42-44]. It has been reported that IL-6 could protect tumors from chemotherapy-induced apoptosis by regulating the expression of apoptosis-related genes [32, 45]. Whether the SDF-1α-induced IL-6 autocrine loop regulates the expression of apoptosis-related genes in PCCs needs further investigation.

The SDF-1α/CXCR4 biological axis can activate multiple intracellular signaling pathways, including the FAK, AKT, ERK1/2, P38, STAT3, mTOR, and Wnt/β-catenin signaling pathways in various cancers [24, 35, 46, 47]. Our results indicated that the FAK-AKT, ERK1/2 and P38 signaling pathways were all activated by SDF-1α in PCCs, and the FAK-AKT and ERK1/2 signaling pathways most likely mediated the SDF-1α-induced upregulation of IL-6 in PCCs. Consistent with the present study, our previous findings also demonstrated the critical role of FAK-AKT in the ECM-induced chemoresistance of PCCs, and the combination of selective FAK inhibitors with GEM might be a very promising therapeutic strategy for pancreatic cancer [11]. These results suggested that FAK-AKT and ERK1/2 might be critical intracellular signaling pathways in PCCs through mediation of the tumor-stroma interaction. The selective inhibition of these signaling pathways may present a potential therapeutic target for pancreatic cancer.

In conclusion, our results demonstrate that PSCs contribute to the chemoresistance of PCCs to GEM. Moreover, we show for the first time that the effects of PSCs on GEM chemoresistance are mediated through paracrine SDF-1α/CXCR4 signaling and, subsequently, the IL-6 autocrine loop in PCCs. Our results also reveal that SDF-1α-induced IL-6 upregulation in PCCs may be partially due to its downstream activation of the intracellular FAK-AKT and ERK1/2 signaling pathways. Our findings indicate that the interaction between PSCs and PCCs plays a critical role in pancreatic cancer chemoresistance, and blocking the PSC-PCC interaction by inhibiting the SDF-1α/CXCR4 axis and its downstream signaling pathways may be a promising therapeutic strategy for overcoming chemoresistance in pancreatic cancer.

## MATERIALS AND METHODS

### Cell culture and isolation

Human PCC lines (MIA PaCa-2, Panc-1, AsPC-1, BxPC-3) were purchased from the American Type Culture Collection (ATCC). The cell lines were maintained in culture as an adherent monolayer in RPMI-1640 or Dulbecco's Modified Eagle's Medium (DMEM) supplemented with 10% fetal bovine serum (FBS) and 100 mM each of penicillin and streptomycin. Cells were grown at 37°C with 5% CO2 in a humidified atmosphere.

Human PSCs were isolated from four surgically resected pancreatic cancer tissues as previously reported (Bachem et al, 1998), and the clinicopathological data of the four patients are listed in Table 1. Briefly, tumor tissues were cut into 1-mm3 cubic blocks with a sterile sharp blade and seeded in 6-well culture plates with 4 to 6 pieces per well. The explants were kept in DMEM/F-12 (1:1) culture medium containing 10% FBS with antibiotics, and the medium was changed every 2 days. After cells grew out from the tissue explants and reached 90% confluence, the cells were digested with 0.05% trypsin and cultured in DMEM/F-12 with 10% FBS at 37°C and 5% CO_2_. PSCs from passages 3 to 7 were identified by immunofluorescence staining for α-SMA (Dako, Carpenteria, CA, USA) and vimentin (Abcam, Cambridge, MA, USA) and used for further study. All human samples were obtained in accordance with the policies and practices of the Institutional Review Board of Peking Union Medical College Hospital.

**Table 1:** The clinicopathological data of four patients with surgically resected pancreatic cancer

Number	Gender	Age	Differentiation	Size(cm)	Location	TNM stage Grade
S1	F	71	Moderate	6	Body	IIA (T3N0M0)
S2	M	55	Moderate	3	Tail	IIA (T3N0M0)
S3	F	57	Well	5	Tail	III (T4N0M0)
S4	F	80	Well	3	Head	IIB (T3N1M0)

### Preparation of conditioned medium

Primary PSCs (1×10^6^ cells) were plated in T25 flasks containing FBS-free DMEM/F12 (1:1). After 24 h, the PSC-conditioned medium (PSC-CM) was harvested, centrifuged at 1,200 rpm for 5 min, and stored at −80°C until use. For the indirect co-culture with PCC, PSC-CM was added to the tumor cells, and the medium was changed every day for 3 days.

### Reagents

Gemcitabine (GEM) was purchased from Eli Lilly (Indianapolis, IN, USA). AMD3100, MTT, Hoechst 33342, PD98059 (an ERK inhibitor), PF573228 (a FAK inhibitor), SB20938 (a P38 inhibitor) were all supplied by Sigma-Aldrich (St. Louis, MO, USA). Recombinant human SDF-1α (rhSDF-1α), the anti-IL-6 mouse monoclonal antibody (mAb) and the IL-6 ELISA kit were purchased from R&D Systems (Minneapolis, MN, USA). The anti-αSMA mouse mAb was from Dako. The anti-vimentin and anti-SDF-1α mouse mAbs, the anti-CXCR4 rabbit polyclonal antibody and the SDF-1α neutralizing antibody (SDF-1α Ab) were from Abcam. Rabbit mAbs against pAKT (Ser473), pP38 (Thr180/Tyr182), ERK1/2, pERK1/2 (Thr202/Tyr204) and pFAK (Tyr397) were from Cell Signaling Technologies (Beverly, MA, USA). The mouse mAb against FAK, rabbit mAbs against AKT and and β-actin, the rabbit polyclonal antibody against P38, and the secondary horseradish peroxidase-conjugated anti-rabbit and anti-mouse antibodies were all purchased from Santa Cruz Biotechnology (Santa Cruz, CA, USA).

### Evaluation of gene expression by real-time PCR (RT-qPCR)

Total RNA was extracted from PCC and PSC using TRIzol reagent (Invitrogen, Carlsbad, CA, USA). The complementary DNA was synthesized using the High Capacity cDNA Reverse Transcription Kit (Applied Biosystems, Foster City, CA, USA) according to the manufacturer's protocol. RT-qPCR was performed as described by the manufacturer using the Power SYBR green PCR master mix (Applied Biosystems). The sequences of the primers used are listed in Table 2. The transcript level for each specific gene was normalized to the level of β-actin mRNA and was calculated using the comparative threshold cycle (Ct) method (2^−ΔΔCt^).

**Table 2:** List of primers

Name	Forward	Reverse	Production size	Ta Opt °C
β-actin	GCCAACACAGTGCTGTCTGG	GCTCAGGAGGAGCAATGATCTTG	100 bp	50.4
SDF-1α	TCCCAGCTATTCCTACTCTCTC	CCAGCAATCACCCTCTTC	114 bp	52.6
CXCR4	GAAATCATCAAGCAAGGGTG	GCTCCAAGGAAAGCATAGAG	119 bp	56.0
IL-6	GTCCAGTTGCCTTCTCCC	GCCTCTTTGCTGCTTTCA	223 bp	53.7
IL-8	TCCAAACCTTTCCACCCC	CACAACCCTCTGCACCCA	147 bp	51.8

### Immunohistochemistry

Paraffin-embedded tissue sections were deparaffinized, rehydrated, rinsed, immersed in 10 mM sodium citrate, heated for 20 minutes, and cooled for 20 minutes. The sections were incubated with a primary antibody against α-SMA (1:50 dilution), SDF-1α (1:100 dilution), or CXCR4 (1:100 dilution) at 4°C overnight followed by incubation with the secondary antibody. The immunostaining was developed with 3,3′-diaminobenzidine (DAB). Appropriate positive and negative controls were run.

### Western blot analysis

Cells were processed for protein extraction and western blotting using standard procedures. Briefly, the cells were washed twice with phosphate-buffered saline (PBS) and scraped into RIPA lysis buffer containing protease and phosphatase inhibitors. The cell lysates were passed through a needle syringe to facilitate the disruption of the cell membranes and were centrifuged at 14000 rpm for 10 min at 4°C, and the supernatants were collected. Proteins (15 – 30 μg) were resolved by electrophoresis on 10-15% SDS-PAGE gels, transferred onto polyvinylidene difluoride membranes and subjected to a standard immunodetection procedure using specific antibodies against ERK1/2, pERK1/2, AKT, pAKT, FAK, pFAK, P38, pP38 and β-actin (1:1000 dilution). All secondary antibodies were used at a 1:5000 dilution. The blots were processed with the ECL Plus Western Blotting detection kit (Pierce Biotechnology, Rockford, IL, USA), and the signal was detected using an LAS-3000 image analyzer (Fuji Photo Film Co., Tokyo, Japan).

### Cell viability assay

Panc-1 cells were seeded in 96-well plates at a density of 4000 cells per well and allowed to settle for 24 h, followed by treatment with GEM (10 μM) under serum-free conditions in the presence or absence of various doses of PSC-CM (20%, 40%, 60%, 80%, and 100%) and SDF-1α Ab. After 24 h, 48 h, or 72 h of treatment, cell growth was determined using the MTT cell proliferation assay. Viability was calculated as percent (%)=(A/B) x100, where A and B were the absorbance of the experimental and control groups, respectively.

### Analysis of apoptosis based on nuclear morphology

Distilled slides were placed onto the surface of 6-well plates. Panc-1 cells (1.6×10^5^) were seeded onto the slides, allowed to settle for 24 h in serum-free medium, and then cultured with fresh medium containing GEM (10 μM) in the presence or absence of PSC-CM, rhSDF-1α (100 ng/ml), SDF-1α Ab (100 ng/ml) and/or IL-6 Ab (1 μg/ml) for 72 h. After treatment, the slides were washed with PBS, and the cells were fixed with 4% paraformaldehyde for 20 min. The slides were washed again with PBS, and 0.1 ml of Hoechst 33342 (1 μg/ml) was added to each slide. The slides were incubated in the dark at room temperature for 15 min and then washed three times with PBS. The cells were examined using a fluorescence microscope (Motic, Germany) and photographed. Apoptosis was evaluated based on nuclear condensation.

### Flow cytometric assay of apoptosis

Panc-1 cells were treated as described in Section 2.8. The cells were collected, washed twice in ice-cold PBS, resuspended in 1× binding buffer, and then incubated with FITC-conjugated annexin V and PI (BD Pharmingen, Franklin Lakes, NJ, USA) for 15 min at room temperature in the dark. The samples were analyzed by FACS using Cell Quest Research Software (BD Pharmingen).

### Enzyme-linked immunosorbent assay

Panc-1 cells (1.6×105) were seeded in 6-well plates and cultured in serum-free medium for 24 h. The cells were then cultured with fresh medium for 24 h, 48 h or 72 h. The culture supernatants were collected, centrifuged at 1500 r.p.m for 5 min to remove particulate material, and frozen at −80°C until use. The cell number in each well was counted. IL-6 was measured using an IL-6 ELISA Kit according to the manufacturer's instructions. The IL-6 concentration was normalized to 1×105 cells in each sample.

### Statistical analysis

Each experiment was performed at least three times, and all the values were expressed as the mean±SE. The differences between the groups were compared using student's t-tests. The level of significance was defined as *P*≤0.05 (two tailed).

## Abbreviations

CXCR4: CXC chemokine receptor 4
ECM: Extracellular matrix
EMT: Epithelial-mesenchymal transformation
GEM: Gemcitabine
IL-6R: IL-6 receptor
MMP: Matrix metallopeptidases
PCC: Pancreatic cancer cell
PSC: Pancreatic stellate cell
PSC-CM: PSC-conditioned medium
SDF-1: Stromal cell-derived factor-1
